# Fast Adaptive RNN Encoder–Decoder for Anomaly Detection in SMD Assembly Machine

**DOI:** 10.3390/s18103573

**Published:** 2018-10-22

**Authors:** YeongHyeon Park, Il Dong Yun

**Affiliations:** Department of Computer and Electronic Systems Engineering, Hankuk University of Foreign Studies, Yongin 17035, Korea; yeonghyeon@hufs.ac.kr

**Keywords:** anomaly detection, fast adaptation, RNN encoder–decoder

## Abstract

Surface Mounted Device (SMD) assembly machine manufactures various products on a flexible manufacturing line. An anomaly detection model that can adapt to the various manufacturing environments very fast is required. In this paper, we proposed a fast adaptive anomaly detection model based on a Recurrent Neural Network (RNN) Encoder–Decoder with operating machine sounds. RNN Encoder–Decoder has a structure very similar to Auto-Encoder (AE), but the former has significantly reduced parameters compared to the latter because of its rolled structure. Thus, the RNN Encoder–Decoder only requires a short training process for fast adaptation. The anomaly detection model decides abnormality based on Euclidean distance between generated sequences and observed sequence from machine sounds. Experimental evaluation was conducted on a set of dataset from the SMD assembly machine. Results showed cutting-edge performance with fast adaptation.

## 1. Introduction

Surface Mounted Device (SMD) assembly machine manufactures products by processing a series of sequential operations at a high-speed and changes target products frequently. Monitoring in anomaly detection is essential for SMD assembly machines. Anomaly detection is a very challenging problem. Each operation is very short; therefore, it can be confusing to distinguish. Table 1 shows a daily record of product manufacturing. Each product is manufactured in small quantities and switching to a new target-product requires a preparing time. It is very useful if an anomaly detection model can respond quickly within the preparing time. In this paper, we proposed an anomaly detection model based on Recurrent Neural Network (RNN) Encoder–Decoder [1,2]. Besides detecting anomalies, we also focused on ensuring fast adaptation to the frequent product-switching process. Sound information of the operating machine is preferred for training the model due to the high speed of the manufacturing process.

Previous studies have described various sound-based approaches to solve similar problems using a classification model such as Support Vector Machine (SVM) and Convolutional Neural Network (CNN) [3,4,5,6,7]. These studies solved the problem by classifying states of anomalies and achieved very high performance in classifying known classes. SVM and CNN requires collection of all kinds of normal and abnormal data for training because they are supervised learning models. However, collecting all kinds of data is practically impossible because some abnormal states are not expressed. Therefore, SVM and CNN would cause misclassification problem due to the high probability that a novel normal state is classified as abnormal and novel abnormal vice versa.

However, it is possible to collect normal state data from the SMD assembly machine. The unsupervised learning model can be trained with only normal state data without class distinction [8,9]. Oh and Yun [10] have solved the anomaly detection problem in SMD assembly machine and achieved high performance with an Auto-Encoder (AE) based model. However, it requires a huge number of parameters (neuron of the neural network) and several hours for training. Thus, it is difficult to apply such method to the actual field because the training time is longer than the average preparation time shown in Table 1. Pre-training with normal state data for all SMD products can be considered. Unfortunately, it is impossible to collect the normal state data from specific SMD that has not been through the manufacturing process.

To solve this issue, a generative model with fewer parameters is required other than AE, to train in a short time. RNN has a rolled structure and it unrolls when deriving the sequential output from the sequential input. Unrolling means computing the output using one RNN cell by chaining with another cell. Thus, it is possible to process a large amount of data with a few parameters. The proposed model can be trained within a significantly shorter time to be applicable during the preparing period. The model that we proposed is much more appropriate for solving the anomaly detection problem in a flexible manufacturing process. We named the proposed model as a Fast Adaptive RNN Encoder–Decoder (FARED). The Detail of FARED is given in Section 3.

This paper is organized as follows. First, we presented data preprocessing in Section 2. In Section 3, we explained the structure of FARED. We presented experimental results in Section 4. We concluded this work in the Section 5.

## 2. Data Preprocessing

FARED uses sequential data from operating sounds of the SMD assembly machine as input. It can use raw sound data without any preprocessing. However, we used preprocessed data. This is because using raw data not only needs additional parameters for feature extraction, but also extra training time. However, both training time and the number of parameters can be reduced with the preprocessing technique. We used two types of preprocessing techniques. One was Short-Time Fourier Transform (STFT) and the other was Mel-Frequency Cepstral Coefficients (MFCC) feature extraction.

The MFCC feature extraction is very efficient in speech recognition [11,12]. Because it can extract frequency information from the spectrum and reduces dimension of data, dimension reduction will decrease the amount of computation cost and training time. We also used STFT for comparing the performance at anomaly detection with MFCC feature extraction considering lost detailed information of the spectrum with dimensional reduction by MFCC feature extraction. We assumed that shorter training time would be used with MFCC feature extraction rather than STFT.

**Figure 1:**
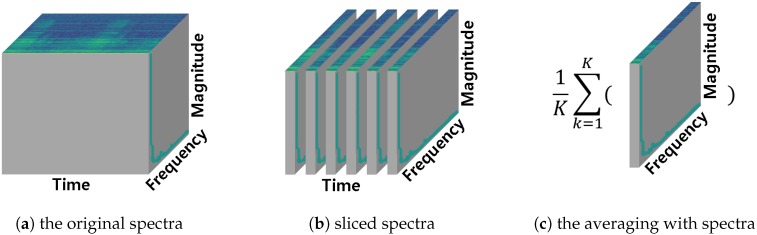
The process for obtaining the average spectrum of spectra. (**a**) original spectra obtained from Short-Time Fourier Transform (STFT) or Mel-Frequency Cepstral Coefficients (MFCC) feature extraction; (**b**) sliced by time axis spectra according to the number of window used in STFT or MFCC feature extraction; (**c**) average of sliced spectra.

**Figure 2:**
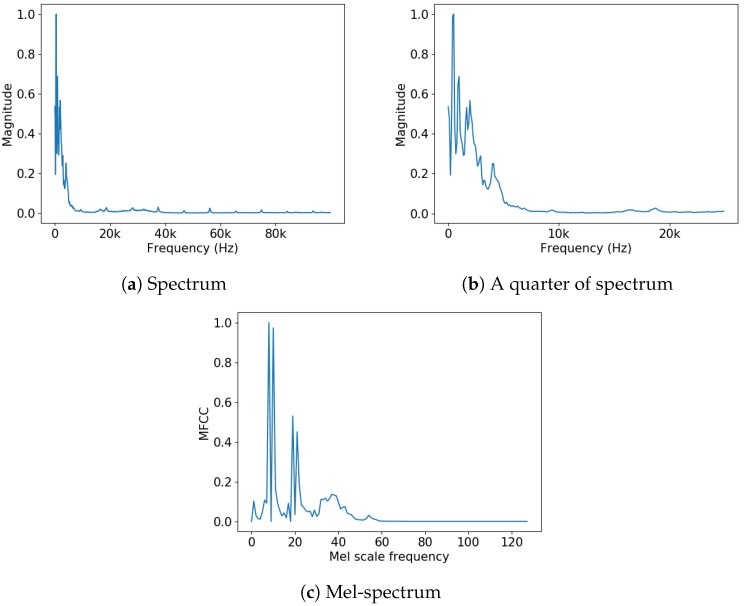
Subfigures (**a**,**b**) are time-averaged spectrum after STFT and (**c**) is 128-dimensional time-averaged Mel-spectrum after MFCC feature extraction. (**a**) represents a range of 96 kHz, with a resolution of 1025; (**b**) is an expanded view of a quarter of (**a**). Each unit of (**c**) represents one MFCC filter bank.

First, we sliced the entire sound data in 500 ms with 50% overlapping. It helps learning the data easily for the model because it provides redundant information between each sequence. After slicing, we applied STFT and MFCC feature extraction respectively to each slice. We used the Hann window for windowing, 2048 for window length, and 512 for hop length in STFT. Each spectra consists of spectrum $\vec{s_{1}}$ to spectrum $\vec{s_{k}}$ after STFT or MFCC feature extraction. The symbol *k* means the number of window used in preprocessing. Finally, we calculated a time-averaged spectrum $\vec{x}$ using Equation (1) and normalized the range of magnitude from 0 to 1. The procedure of obtaining time-averaged spectrum is shown in Figure 1 and sample of preprocessed data is shown in Figure 2:

(1) $$\vec{x}=\frac{1}{K}\sum_{k=1}^{K}\vec{s_{k}}.$$

The spectrum of spectra obtained after preprocessing can be used directly. However, we used a time-averaged spectrum because it could ease minute noise generated during machine operation. Therefore, we used a time-averaged spectrum to construct input and ground truth for FARED. We refer to the spectrum for construct input data as $\vec{x}$ and output spectrum of FARED as $\vec{y}$. Dimensions of the $\vec{x}$ and $\vec{y}$ were the same as 192 k, 1025 and 128 for raw data, spectrum from STFT and MFCC feature extraction, respectively. We used data preprocessed by STFT and MFCC feature extraction for experiments.

## 3. Fast Adaptive RNN Encoder–Decoder

In this section, we will present the architecture of FARED based on RNN Encoder–Decoder. We used Long-Short Term Memory (LSTM) for each RNN cell. We will also describe the training algorithm and how to calculate the number of parameters of FARED.

### 3.1. RNN Encoder–Decoder Based Architecture

**Figure 3:**
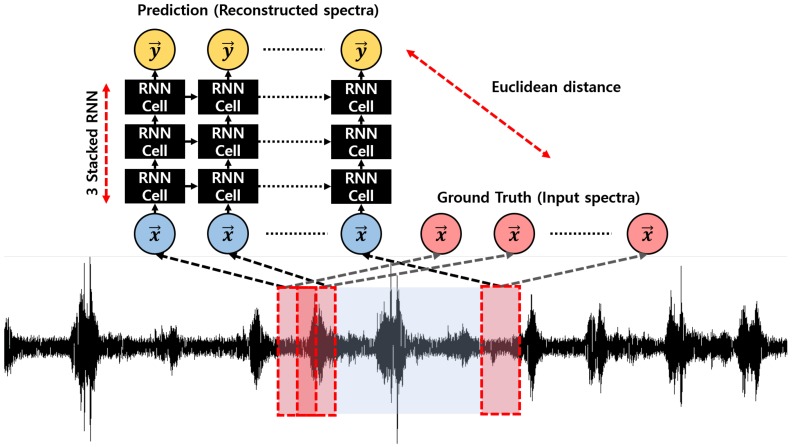
Structure of Fast Adaptive Recurrent Neural Network (RNN) Encoder–Decoder (FARED). The input is constructed by sequential spectrum (red box). Each spectrum has 50% of overlapping in the time domain. The output is a sequential reconstructed spectrum from input sequences. The Euclidean distance between prediction and ground truth is used for training and anomaly detection.

RNN Encoder–Decoder is one of the generative models generally used for machinet translation [1,2]. FARED is designed for reconstructing input sequences as shown in Figure 3. The learning method in the form of conditional probability and generative model already given for FARED is shown as follows:

(2) p(Y|X)∝p(X|Y)p(Y).

In Equation (2), X and Y are a set of sequential input and output spectrum ($\vec{x}$ and $\vec{y}$), respectively. The p(Y|X) is the posterior score (uncertainty), p(X|Y) is likelihood, and p(Y) is prior knowledge. We have information of prior knowledge and posterior score from the training data. Therefore, the information that we want to know is likelihood. It will be learned by the maximum likelihood estimation in the training process.

FARED based on RNN Encoder–Decoder is presented in Figure 3. Some research studies have used the model for predicting future sequence from current sequence [13,14]. We refer to the model used in a previous research, ‘Ref-RED’. FARED is a modified model of Ref-RED. When Ref-RED is used to predict repeated future sequence, it works well. For example, Ref-RED works well to anomaly detection in Electrocardiography (ECG) because the pattern of ECG has one-to-one correspondence. For example, only *R* wave appears after *Q* wave in normal ECG. The SMD assembly machine has one-to-many correspondence that makes it difficult to learn and predict future sequence. Therefore, we modified Ref-RED to FARED to restore the current sequence like conventional AE [8,9] because it is easy to learn by restoring the current sequence rather than predicting future sequence. We need a fast adaptation model for the manufacturing environment. Thus, we need to find a model that learns data easier and faster. We have performed experiments to compare multiple REDs for finding the better model [15]. In that experiment, we confirmed that a structure like FARED could learn the data more effectively than others.

To detect anomalies in real time, it is necessary to decide if the input is abnormal or not in a short period. FARED also has shorter decision time than Ref-RED. Assuming that the length of the input and output sequence are equal to *L*, Ref-RED decides anomaly or not in 2L times because it needs to observe the input sequence and the next *L* sequences for decision. However, FARED needs only *L* time for decision. This means that FARED is a more efficient model. FARED, the model that we proposed, can be called, ‘Fast Adaptive’. We will show that FARED is ‘Fast Adaptive’ through experiments in Section 4.3.

We trained FARED to learn the likelihood with only normal state data, so it can generate input sequences well when input sequences were normal state sequences. However, if the sequence of the input data was shuffled or not observed in the training data, FARED could not function well due to the low likelihood that the model knew. The training algorithm is presented in Algorithm 1. The input data X consists of *p* sequential spectrum while the output data Y also has the same length of sequential spectrum. Sequential vectors are obtained after preprocessing as shown in Section 2. We initialized parameters of the neural network using Xavier initializer [16]. The purpose of Algorithm 1 is to minimize the Euclidean distance between X and Y. We used RMSprop optimizer for minimizing [17].

**Algorithm 1** Training algorithm for RNN Encoder–Decoder **Input: Set of the sequential input spectrum**
$X=\{{\vec{x}}_{1},{\vec{x}}_{2},{\vec{x}}_{3}\cdots {\vec{x}}_{p}\}$ **Output: Set of the sequential output spectrum**
$Y=\{{\vec{y}}_{1},{\vec{y}}_{2},{\vec{y}}_{3}\cdots {\vec{y}}_{p}\}$ Initialize network parameters by Xavier initializer **while** the loss has not converged **do**  Compute loss between X and Y using Euclidean distance [16]  Update parameters by RMSprop optimizer [17] **end while**

### 3.2. Long-Short Term Memory

In the RNN Encoder–Decoder, the RNN cell can be selectively used in vanilla RNN [18], Long- Short Term Memory (LSTM) [19], and Gated Recurrent Unit (GRU) [20]. We used LSTM for constructing the model. This is because vanilla RNN has vanishing gradient problem when the length of input or output sequences becomes long. We can also consider using GRU. However, Chung et al. [21] have already reported that there is no superiority between LSTM and GRU. Therefore, we used LSTM for constructing FARED. Each LSTM cell has three types of gates and two types of states as follows:

(3) $${\vec{i}}_{t}=\sigma \left(W_{i}{\vec{x}}_{t}+U_{i}{\vec{h}}_{t-1}+b_{i}\right),$$

(4) $${\vec{o}}_{t}=\sigma \left(W_{o}{\vec{x}}_{t}+U_{o}{\vec{h}}_{t-1}+b_{o}\right),$$

(5) $${\vec{f}}_{t}=\sigma \left(W_{f}{\vec{x}}_{t}+U_{f}{\vec{h}}_{t-1}+b_{f}\right),$$

(6) $${\vec{c}}_{t}={\vec{f}}_{t}\circ {\vec{c}}_{t-1}+{\vec{i}}_{t}\circ tanh\left(W_{c}{\vec{x}}_{t}+U_{c}{\vec{h}}_{t-1}+b_{c}\right),$$

(7) $${\vec{h}}_{t}={\vec{o}}_{t}\circ tanh\left({\vec{c}}_{t}\right).$$

**Figure 4:**
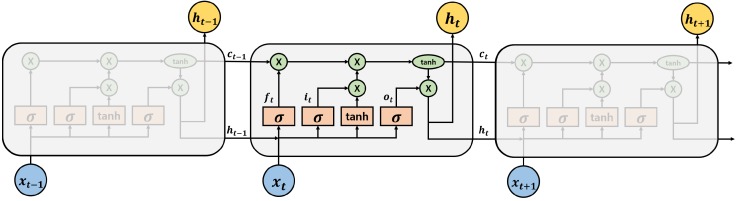
The structure of the Long-Short Term Memory (LSTM) cell. It contains input gate ${\vec{i}}_{t}$, output gate, ${\vec{o}}_{t}$, forget gate ${\vec{f}}_{t}$, hidden state ${\vec{h}}_{t}$ and cell state ${\vec{c}}_{t}$. The σ symbol means sigmoid function and tanh means hyperbolic tangent function.

Equations (3) to (5) present three types of gates. Two types of states are shown in Equations (6) and (7). For each equation, ${\vec{x}}_{t}$ is the input vector (spectrum), ${\vec{c}}_{t}$ is cell state vector, and ${\vec{h}}_{t}$ is the hidden state vector of the LSTM. ${\vec{h}}_{t}$ is the same as the output spectrum ${\vec{y}}_{t}$, and each *W*, *U*, *b* of equations are the parameter of the LSTM. Forget gate, input gate and output gate’s activated vectors are ${\vec{f}}_{t}$, ${\vec{i}}_{t}$, ${\vec{o}}_{t}$ respectively. The ∘ symbol in Equations (6) and (7) means Hadamard products. Figure 4 shows the structure of the LSTM and it contains all of the equations. Equations and Figure 4 show that LSTM derives output with causality. RNN derives output using the same parameter for every divided input. Thus, RNN can process the same size of data with a smaller number of parameters than AE.

We can calculate the number of parameters *P* of the proposed model with dimension of the cell state ($n_{c}$), input ($n_{i}$) and output ($n_{o}$) as follows [22]:

(8) $$P=4n_{c}n_{c}+4n_{i}n_{c}+n_{c}n_{o}+3n_{c}.$$

The proposed model is constructed by three stacked LSTM and its input dimension is 128 when using the MFCC feature extraction shown in Figure 2. Table 2 shows the number of parameters *P* of two models. AE by Oh and Yun [10] has about 11 times as many parameters than FARED that means AE requires more time to train the parameters than FARED and more computational resources.

## 4. Experiments

In this section, we will explain how we acquire the dataset. We also compared and confirmed the performance of FARED in anomaly detection as well as fast adaptation with previous works.

### 4.1. Dataset

**Figure 5:**
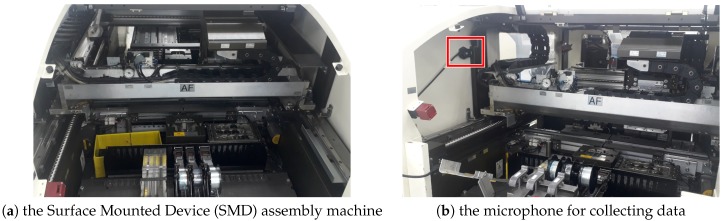
The SMD assembly machine with microphone. The microphone that is attached as shown in (**b**) is used for collecting sound data when the machine is operating.

The dataset (CREVIS Co., Ltd.,Yongin, Korea) consisted of sound data acquired from SMD assembly machine with 192 kHz of sampling rate. The data collection process is shown in Figure 5. Sequential machine operational sound data were collected from an operating SMD assembly machine placing a microphone as indicated by the red bounding box in Figure 5b.

The collected dataset is summarized in Table 3. Sample data and source code are available at the Github repository [23]. We experimented with one of the data collected from each manufacturing process as normal while the others were abnormal. This meant that all manufacturing processes were made abnormal except for itself.

We used another dataset from previous research of Oh and Yun [10] to confirm that FARED could detect anomalies well as shown in Table 4. One cycle in each class in Table 4 is equal to 20 s because it belongs to the same manufacturing process. We refer to the dataset in Table 3 as ‘Set-A’ and that in Table 4 as ‘Set-B’.

The whole data of Set-B were collected from the same SMD production. Abnormal state data were ‘Intermittent noise’ and ‘Non-greased’. The ‘Intermittent noise’ class contained ‘Air ejection’, an action to remove the foreign substance in the machine, and ‘Artifact’, a clacking sound made by human. The ‘Non-greased’ class was collected when the machine operated without grease.

### 4.2. Comparison of Preprocessing Methods

**Figure 6:**
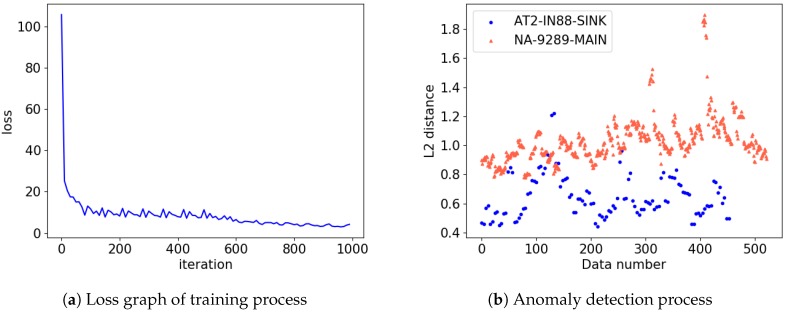
Results of the training and detection process. When GTX 1060 3 GB for 1 k iteration with minibatch size 100 was used, it took about three minutes when using MFCC. The ’NA-9289-MAIN’ is regarded as abnormal while ‘AT2-IN88-SINK’ is regarded as normal. FARED can distinguish between ‘AT2-IN88-SINK’ and ‘NA-9289-MAIN’ as shown in subplot (**b**).

In this section, we compared two types of data preprocessing technique with Set-A in order to construct faster adaptive architecture. We trained FARED with STFT and MFCC feature extraction respectively and compared them to determine which preprocessing technique was more appropriate for FARED. Sequences from only one SMD product were regarded as normal while others were considered as abnormal. We used normal state data for training. Results of training FARED are presented in Figure 6. Seven cycles for each class were performed separately for training. The rest of testing was then performed. The input data with a set of 30 sequences was constructed. The output had the same shape as the input. One sequence contained 500 ms information of sound data and 50% overlapping was applied when slicing the whole sound data. Thus, 30 sequences hold 8 s of information. When using the data preprocessed by STFT, it took about 25 min for training. However, MFCC took only about 3 min because of data dimension. Compared to AE based model proposed by Oh and Yun [10] that took 8 h for training with a set of 32 sequences, 25 min were relatively short. Given preparation time in Table 1, STFT is not appropriate for preprocessing. We also found that 192k-dimensional raw data larger than STFT were not appropriate for usage. MFCC can be preferably trained and used in near real time because of the smaller dimension of the data and reduced a number of parameters.

We measured the Area Under the Curve (AUC) from the Receiver Operating Characteristic (ROC) curve as an indicator of performance [24]. We constructed the ROC curve with lowest to highest reconstruction error as thresholds. In addition, it is possible to find the adequate threshold for filtering out anomalies for each normal state in ROC curve. Results of AUC are shown in Table 5. The AUC is closer to 1 when the model filters out anomalies effectively. It is closer to 0 otherwise.

The performance of FARED when using MFCC is lower than that when using STFT. However, MFCC has an advantage such as reduction of training time for about one-tenth than using STFT because of decreased data dimension from 1025 to 128. This was why MFCC feature extraction was used in our model for fast adaptation. If sufficient preparation time is given, we may consider using STFT.

### 4.3. Fast Adaptive Architecture

**Figure 7:**
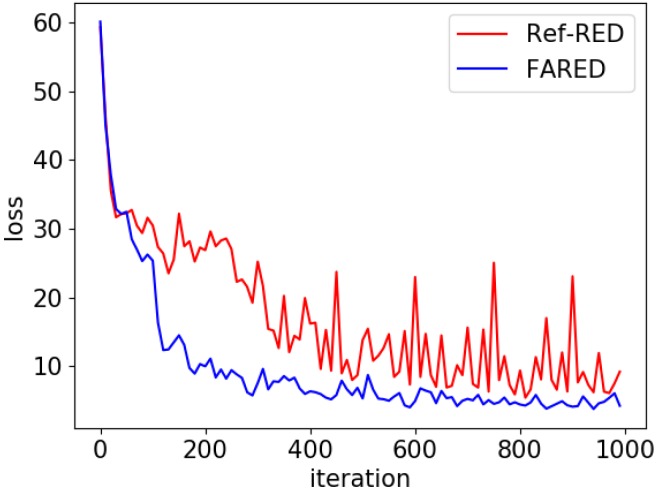
Comparison of Ref-RED and FARED training process. It shows that loss of the FARED converges faster and more stable than Ref-RED under same conditions (1 k iterations with minibatch size 100 of training and 0.005 of learning rate).

The fast-adaptive architecture is shown in Section 3.1. In this section, we confirmed that FARED could adapt faster than Ref-RED with Set-B. We show how faster FARED can adapt to various environments for anomaly detection than previous RNN Encoder–Decoder architecture. We will also compare the anomaly detection performance and the number of parameters with AE used by Oh and Yun [10]. We separated seven cycles of each class for training and the rest for testing. We trained ‘Normal’ class and tested others for detecting anomalies. Results of anomaly detection are shown in Figure 7 and Table 6.

Figure 7 shows that the loss of FARED converges faster than Ref-RED. Since fast adaptation is the most important issue in this research, FARED is more appropriate than Ref-RED. Moreover, the scale of oscillation is significantly reduced because FARED has more stability and reliability.

The average AUC of FARED is the best among three generative models as shown in Table 6. We experimented Ref-RED and FARED in the same conditions such as learning rate, training iterations, and the number of parameters as those used in a previous work [15]. However, FARED is 0.118 (16%) higher than the average AUC of Ref-RED for anomaly detection. This means that FARED adapts faster for detecting anomalies than Ref-RED in the manufacturing environment. That was the reason why we called the architecture ‘Fast Adaptive’.

Furthermore, we achieved higher performance on anomaly detection by using FARED than AE based architecture. Oh and Yun have achieved AUC of 0.980 at ‘Intermittent noise’ and 0.640 at ‘Non-greased’. FARED had lower AUC at ‘Intermittent noise’ than AE, but much better at ‘Non-greased’. The reason for detecting ‘Non-greased’ much better than ‘Intermittent noise’ using FARED is that ‘Non-greased’ set has persistent abnormality while ‘Intermittent noise’ does not have persistent abnormality. ‘Intermittent noise’ has almost the same features as normal operation except for a specific abnormal section.

## 5. Conclusions

In the actual field, the manufacturing process is very flexible because of small quantity batch production. Therefore, in varied environments like ours, it is practically difficult to use the previous anomaly detection model based on AE because they require huge storage resources and long training time.

However, our model, FARED, has the ability of fast adaptation that AE or Ref-RED does not have. FARED can adapt in three minutes for the new manufacturing process. It only needs small computational resources. The reason why training time and computational resource were reduced was due to the structure of FARED and data preprocessing.

In the future, we need to find a compromise preprocessing technique between STFT and MFCC feature extraction for better performance of anomaly detection. To make our model more useful, we also need to classify anomalies. Currently, our model FATED is an unsupervised learning model that cannot define the kind of anomaly is. An ensemble with a classification model that can limitedly classify abnormal states might be needed.

## Figures and Tables

**Table 1:** Daily record of the manufacturing process on 24 April 2018.

Product Name	Production Amount	Preparing Time (min:s)	Cycle Time (s)	Running Time (min)
CT-C134-BOT	50	-	27	25
CT-C134-TOP	50	4:27	30	37
ST-3118	30	10:41	17	36
ST-4214-GE	20	9:24	12	28
NA-9473	60	8:40	13	57
M-3808	8	30:11	15	20
M-3708	8	10:09	13	45
M-4178	8	15:40	24	24
M-4478	8	6:07	18	23
Average	26	10:35	18	32

**Table 2:** Comparison of the number of the parameters.

Model	Auto-Encoder [10]	Fast Adaptive RNN Encoder–Decoder (FARED)
Number of parameters	4,864,160	443,520

**Table 3:** Dataset collected from Surface Mounted Device (SMD) assembly machine (Set-A).

Product Name	Number of Cycle	Cycle Time (s)	Total Time (s)
AT2-IN88-SINK	63	8	527
M-3708	8	13	121
M-4478	8	18	165
NA-9289-MAIN	55	10	581
NA-9473	53	13	696
ST-4214-GE	9	12	114

**Table 4:** Dataset collected from same manufacturing process (Set-B) [10].

Class	Number of Cycle	Cycle Time (s)	Total Time (s)
Intermittent noise	41	20	836
Non-greased	8	20	164
Normal	23	20	470

**Table 5:** Area Under the Curve (AUC) matrix of anomaly detection using Mel-Frequency Cepstral Coefficients (MFCC) feature extraction.

Normal	Short-Time Fourier Transform (STFT)	Mel-Frequency Cepstral Coefficients (MFCC)
AT2-IN88-SINK	0.996	1.000
M-3708	0.992	0.865
M-4478	0.999	0.999
NA-9289-MAIN	0.890	0.712
NA-9473	0.965	0.806
ST-4214-GE	0.978	0.983
Average	0.970	0.894

**Table 6:** AUC of anomaly detection in the same manufacturing process.

Model	Intermittent Noise	Non-Greased	Average
Ref-RED	0.835	0.634	0.735
Auto-Encoder	0.980	0.640	0.810
FARED	0.724	0.983	0.853
